# Regional and local temporal trends in the prevalence of canine heartworm infection in the contiguous United States: 2012–2018

**DOI:** 10.1186/s13071-019-3633-2

**Published:** 2019-07-30

**Authors:** Stella W. Self, Cassan N. Pulaski, Christopher S. McMahan, D. Andrew Brown, Michael J. Yabsley, Jenna R. Gettings

**Affiliations:** 1 School of Mathematical and Statistical Sciences, Clemson University, Clemson, SC 29634, USA; 2 Department of Pathobiological Sciences, School of Veterinary Medicine, Louisiana State University, Baton Rouge, LA 70803 USA; 3 Southeastern Cooperative Wildlife Disease Study, Department of Population Health, College of Veterinary Medicine, University of Georgia, Athens, GA 30602 USA; 4 Warnell School of Forestry and Natural Resources, University of Georgia, Athens, GA 30602 USA

**Keywords:** Heartworm, Temporal trends, USA

## Abstract

**Background:**

Canine heartworm disease is a potentially fatal disease for which treatment is financially burdensome for many pet owners. Prevention is strongly advocated by the veterinary community along with routine testing for infection during annual wellness examinations. Despite the availability of efficacious chemoprophylaxis, recent reports have suggested that the incidence of heartworm disease in domestic dogs is increasing.

**Results:**

Using data from tests for heartworm infection in the USA from January 2012 through September 2018, a Bayesian spatio-temporal binomial regression model was used to estimate the regional and local temporal trends of heartworm infection prevalence. The area with the largest increase in regional prevalence was found in the Lower Mississippi River Valley. Regional prevalence increased throughout the southeastern states and northward into Illinois and Indiana. Local (county-level) prevalence varied across the USA, with increasing prevalence occurring along most of the Atlantic coast, central United States, and western states. Clusters of decreasing prevalence were present along the Mississippi Alluvial Plain (a historically endemic area), Oklahoma and Kansas, and Florida.

**Conclusions:**

Canine heartworm infection prevalence is increasing in much of the USA, both regionally and locally, despite veterinarian recommendations on prevention and testing. Additional steps should be taken to protect dogs, cats and ferrets. Further work is needed to identify the driving factors of the locally decreasing prevalence present along the Mississippi Alluvial plain, Florida, and other areas.

## Background

Over 100,000 dogs in the USA are diagnosed with heartworm infection annually [1]. The development of clinical disease, most commonly manifesting as coughing and exercise intolerance, brings a guarded prognosis [2]. The agent, *Dirofilaria immitis*, is a filarial nematode transmitted by various species of mosquito worldwide [2]. Although domestic dogs and some wild canids (e.g. coyotes, wolves) are the primary hosts, infection has been found in several non-canid species including cats, ferrets and otters [2]. These other infected species are generally considered incidental hosts and rarely develop patent infection. Because this parasite is widespread and can use a diverse number of domestic and wild canids as reservoirs, prevention is currently the best defense to limiting disease in domestic dogs.

Dogs and, to a lesser extent, cats are screened routinely for *D. immitis* infection, providing millions of data points annually for estimating the prevalence of infection over time. Since these data started being reported in the early 2000s, the national prevalence of heartworm infections in dogs has changed little. Bowman and colleagues surveyed clinics throughout the USA and estimated prevalence of 1.4% for the years 2001 through 2007 [3]. Updated data were subsequently analyzed and a prevalence of 1.3% was noted for 2010 through 2012 [4]. The importance of surveillance at regional, state, or even county levels is apparent when comparing national prevalence to state prevalence. In the prior studies, southeastern states were reported to have a prevalence of 3.9% in the former study and 2.9% in the latter. These two studies alone might suggest a stable prevalence, or even a decline, but potential changes in testing practices and availability of tests may have influenced these estimates. More contemporary studies suggest that canine heartworm infection prevalence may be increasing in some areas. Drake and colleagues evaluated data available from the Companion Animal Parasite Council (CAPC) website (http://www.capcvet.org) and found that prevalence appeared to increase between 2013 and 2016 in most of the southeastern states [5]. Other evidence of increasing cases is seen in the 2016 survey conducted by the American Heartworm Society who reported that there was an average increase of 21.7% in the incidence of canine heartworm infection cases per clinic compared to 2013 [6].

As alluded to above, national and state level estimates provide insight on the burden of infection for either the entire canine population or the population within a state. However, the use of administrative borders to aggregate these data is not appropriate for a vector-borne pathogen that is not impacted by these boundaries. Instead, regional estimates should be derived from a smoothing process that is determined by the data [7]. As stated, regional estimates are useful for understanding the broad trends in prevalence, but examination of prevalence estimates at smaller spatial units, e.g. counties, can reveal striking differences in prevalence, even within a single state. For this reason, we also examine locally-derived trends. These are important for mosquito-borne pathogens since small-scale differences in the environment can alter the diversity and density of competent vectors and number of canid reservoirs [8].

**Fig. 1:**
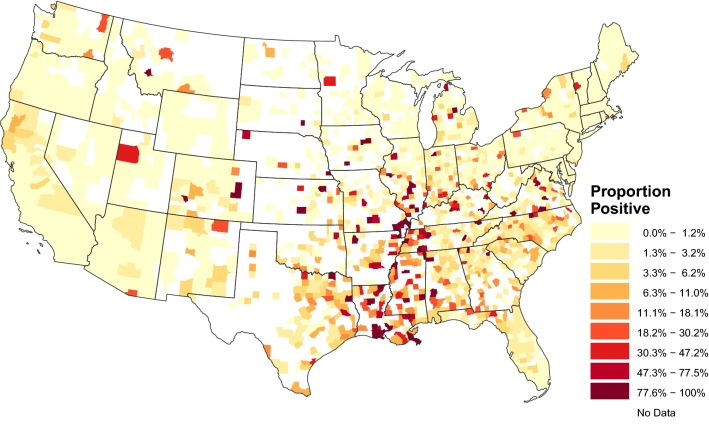
Overall canine heartworm infection prevalence for January 2012 through September 2018. Prevalence is defined as the proportion of positive tests from all tests reported in a county

Heartworm antigen tests performed annually across the USA provide us with data to measure the trends in prevalence over time. Test results are currently provided to the CAPC by IDEXX and ANTECH Laboratories (Fig. 1). Data are available at the monthly and county and county-equivalent level within the contiguous USA from 2012 to present [1]. These trends can highlight areas where prevalence is increasing, and where, therefore, veterinarians and pet owners need to be more vigilant in preventing heartworm infection. There is also growing concern in the veterinary community regarding the existence of macrocyclic lactone resistant strains of the parasite [9], as there is only one drug class currently available to prevent heartworm disease. These trends may help researchers identify possible focal areas of drug resistant infections. Thus, in this study, we aimed to obtain a national picture of the recent changes in heartworm infection prevalence by using a Bayesian spatio-temporal binomial regression model to estimate regional and local temporal trends based on test data from January 2012 through September 2018.

## Methods

### Data

A total of 57,746,055 test results were collected from the point-of-care SNAP^®^4Dx^®^Plus Test, SNAP^®^Heartworm RT Test, and PetChek^®^ from IDEXX Laboratories, Inc. (Westbrook, ME, USA) and Dirochek Assay from ANTECH Laboratories (Fountain Valley, CA, USA), performed in-clinic and at regional reference laboratories between January 2012 and September 2018 [1]. All tests rely on the detection of protein antigens from sexually mature, adult female *D. immitis* worms in the blood of infected dogs. These tests are commonly performed annually during wellness visits and can detect infection as soon as 5 months post-infection. The results from individual tests are collated at each laboratory and aggregate data are provided to the investigators at the county and monthly scale. The reported county corresponds to the veterinary clinic, and in some cases may not be the same as the resident county of the dog. It is reasonable to assume that many of the dogs represented by these data are treated and will subsequently test negative, likely within a year. Therefore, these data provide a better estimate of newly acquired infections compared to antibody-based tests for which a single dog may test positive for multiple years. Figure 1 depicts an aggregation of the heartworm infection data, from January 2012 to September 2018. Displayed are the proportion of positive tests of all tests reported for each county, defined here as prevalence. Counties indicated in white are those that did not report any test results.

### Model definition

The spatio-temporal binomial regression model developed in [10] is adopted to estimate and evaluate local and regional trends of canine heartworm infection. The development and specification of the model discussed here is fully described in Self et al. [10]. To elucidate the salient features of this model, let $Y\left( {s,t} \right)$ denote the number of positive tests in county $s$ at time $t$, with $n\left( {s,t} \right)$ denoting the total number of tests for the same. The resolution considered here is that of monthly data collected at the county level. To model these data, it is assumed that $Y\left( {s,t} \right)$ conditionally obeys a binomial distribution, i.e.

1 $$ Y\left( {s,t} \right)|n\left( {s,t} \right),p\left( {s,t} \right) \sim {\text{Binomial}}\left\{ {n\left( {s,t} \right),p\left( {s,t} \right)} \right\}, $$

where $p\left( {s,t} \right)$ is the prevalence of the disease in county $s$ at time $t$. To evaluate regional trends, we assume that

2 $$ g\left\{ {p\left( {s,t} \right)} \right\} = \eta_{st} = \delta + \beta \left( s \right)t + \xi \left( {s,t} \right), $$

where $\eta_{st}$ is a linear predictor which determines the prevalence $p\left( {s,t} \right)$ *via* a link function $g^{ - 1} \left( \cdot \right) = { \exp }\left( \cdot \right)/\left\{ {1 + { \exp }\left( \cdot \right)} \right\}$; $\delta$ is an intercept parameter; $\beta \left( s \right)$ is a regression coefficient unique to the $s$th county; and $\xi \left( {s,t} \right)$ is a random effect. For computational reasons, time $t$ is re-scaled to the unit interval.

In the model formulation, $\beta \left( s \right)$ represents the regional trend for the $s$th county, with the convention that the event that $\beta \left( s \right)$ is greater than, less than, or equal to zero indicates that the prevalence is increasing, decreasing, or remaining constant in time, respectively. To allow for changes in regional trends across space, $\beta \left( s \right)$ is parameterized so that it can vary smoothly over the study area. To accomplish this task and to borrow information across neighboring geographic areas, a Gaussian predictive process (GPP) was used to model $\beta \left( s \right)$; for further details on GPPs see [11]. The specifications for the GPP used here are identical to those in [10].

It is well known that ignoring spatio-temporal dependence, when present, can lead to both inaccurate estimation and inference. To avoid these issues, we included $\xi \left( {s,t} \right)$ to account for the spatio-temporal dependence in the data. These parameters provide additional flexibility which enhances the model’s ability to distinguish between spatial trends and spatial noise. Following the works of [12] and [13], the $\xi \left( {s,t} \right)$ were specified based on a conditional autoregressive (CAR) model; for more information on CAR models see [7]. In particular, we followed the model specified in Self et al. [10], that is, we assumed $\varvec{\xi}_{1} \sim {\text{Normal}}\left\{ {{\mathbf{0}}, \tau^{2} \left( {\varvec{D} - \rho \varvec{W}} \right)^{ - 1} } \right\}$ and $\varvec{\xi}_{t} \sim {\text{Normal}}\left\{ {\zeta\varvec{\xi}_{t - 1} ,\tau^{2} \left( {\varvec{D} - \rho \varvec{W}} \right)^{ - 1} } \right\}$. Here, $\zeta$ models the degree of temporal correlation, $\rho$ is a propriety parameter, and $\varvec{D}$ and $\varvec{W}$ are matrices encapsulating the spatial adjacency structure between counties. For more on general autoregressive structures, see [14].

To complete parameter estimation and inference, we proceeded *via* the Bayesian paradigm. Diffuse priors are specified for all unknown model parameters. Posterior sampling is facilitated through Markov chain Monte Carlo (MCMC) methods. Based on a posterior sample obtained from the MCMC algorithm, posterior estimation and inference proceeds as usual. The results are provided below and depict the positive and negative temporal trends in canine heartworm infection prevalence from January 2012 to September 2018.

In addition to estimating the regional trends, we also used our model to estimate local trends. The regional trend in each county was estimated using information from a fairly large surrounding area, and encapsulates the general trend seen over a wide area. The local trend at each county was estimated *via* the county’s linear predictor and captures county level deviations from the regional trends. The local trends rely more heavily on county specific information than do the regional trends. For more details on the differences between the two types of trends, see [10]. Let $\eta_{st}^{\left( g \right)}$ denote the value of $\eta_{st}$ calculated using the parameters from the $g$th posterior draw from the MCMC output. For each county $s$ and each MCMC draw $g$, the following ordinary least squares linear model is fit:

$$ \eta_{st}^{\left( g \right)} = \alpha_{0s}^{\left( g \right)} + \alpha_{1s}^{\left( g \right)} t + \epsilon_{st}^{\left( g \right)} ,\quad t = 1, \ldots ,T, $$

where the $\epsilon_{st}^{\left( g \right)} s$ are independent and identically distributed normal errors with mean 0. The set $\left\{ {\alpha_{1s}^{\left( g \right)} } \right\}$ is a sample from the posterior distribution of the local trend from county $s$, $\alpha_{1s}$. This sample can be used to generate point estimates and draw inference in the usual way. Significance is determined as in [10]. For further discussion and complete details on the formulation and implementation of this model see [10].

## Results

**Fig. 2:**
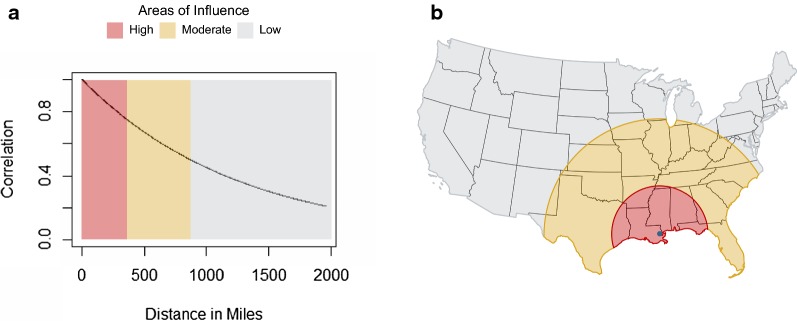
Correlation between the trend parameter for a county and any other county as a function of distance. **a** The curve represents the decaying influence that any other county has on a given county as the distance between them increases. Three distances were chosen arbitrarily for demonstration and are shown here as the three colored boxes, and in (**b**) as circles around a given county. **b** The high, moderate, and low areas of influence for Orleans Parish, LA, are depicted as circles overlaying the prevalence map. Counties within the small circle had a much greater influence on the regional trend estimate for Orleans Parish than those within the low area

The described analysis provides two perspectives of the changing canine heartworm infection prevalence within the contiguous USA: regional and local. The regional trends were estimated for each county by aggregating data from surrounding counties, where the influence of the data from these surrounding counties diminishes with increasing distance. For a formal depiction of how influence decreases with distance, see Fig. 2a. An example for Orleans Parish, LA, is depicted in Fig. 2b. The areas of influence demarcated on the map are for illustration only. Three groups were chosen and designated as high (correlation above 0.75), moderate (correlation between 0.75 and 0.5), and low (correlation less than 0.5) influence. The distances that correspond to these correlation values based on Fig. 2a are 0–361 miles, 362–870 miles, and greater than 870 miles, respectively. In reality, the influence diminishes in a continuous fashion as distance increases as depicted in Fig. 2a.

### Regional temporal trends

**Fig. 3:**
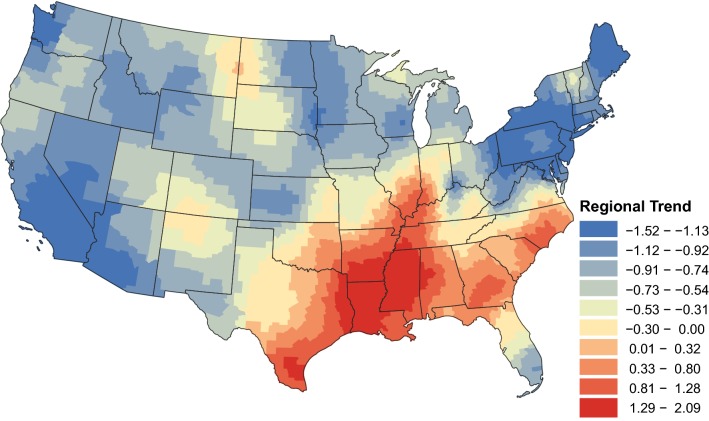
Regional temporal trends 2012–2018: posterior means of the regional temporal trend parameter from Equation (2)

The regional trends describe an increase in prevalence throughout the southern USA. Figure 3 displays the posterior mean of the temporal trend parameter from Equation (2). Positive values indicate an increase in prevalence over time, while negative values indicate a decrease in prevalence. From January 2012 through September 2018, the regional prevalence of heartworm infection in dogs increased in several states in the southern USA, from southern Texas, east to the Atlantic coastline, and up through North Carolina (Fig. 3). Prevalence increased along the Mississippi River as far north as central Illinois. Areas of the greatest increase, as indicated by higher values of the temporal trend parameter, were seen closest to the lower Mississippi river and included areas in Louisiana, Mississippi, southern Arkansas and southwest Tennessee. A notable exception to rising prevalence in the Southeast is Florida. The observed prevalence is lower in Florida compared to nearby states (Fig. 1), and with the exception of the Florida panhandle, no increase in prevalence was evident during the study period (Fig. 3).

### Local temporal trends

**Fig. 4:**
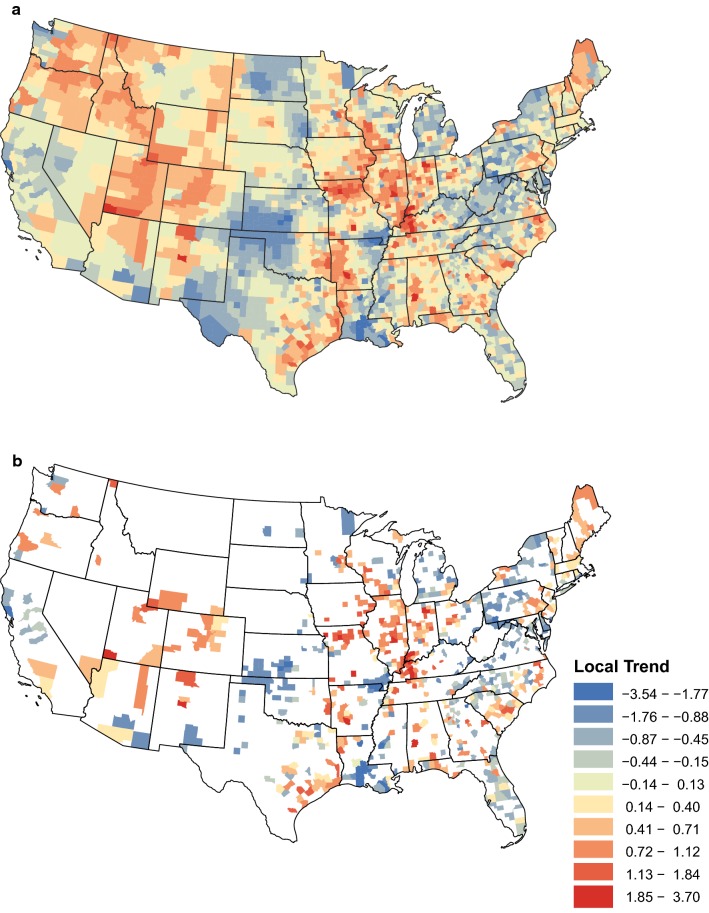
Posterior mean values of the local temporal trend parameter 2012–2018. **a** Posterior means of the local temporal trend parameter for all counties. **b** Posterior means of the local temporal trend parameter for counties in which the 95% credible interval did not contain zero

The second part of the analysis focused on the county-level temporal trends, providing a finer resolution. These results are shown in Fig. 4a. Interpretation of the local trends is best done within the context of statistical significance. Figure 4b shows counties that were statistically different from zero (either above or below) as determined by 95% credible intervals (credible intervals are the Bayesian equivalent of confidence intervals). For counties that report a small number of tests, the ability of the local trends to detect significant changes is more limited. A county’s local trend may be insignificant because there is in fact no significant change occurring in the county. However, a county’s local trend may also be insignificant because there is insufficient testing to detect the underlying change.

The local trends show how much variability was present at the county level, highlighting marked differences even between neighboring counties. Examination of only the significant trends allow us to appreciate some local patterns (Fig. 4b). Along the Atlantic coast, most local trends were increasing, except for areas around the Chesapeake Bay and Long Island Sound. West of the coast throughout the Appalachian region, most local trends were decreasing. Focusing on the central portion of the USA, we can see areas of increasing prevalence with the greatest amount of clustering and largest positive trend values. Most of this area, from eastern Texas to the Appalachian region, experienced increasing prevalence. Michigan and the Upper Midwest are exceptions. Perhaps the most interesting cluster of decreasing prevalence is seen from Louisiana north to southern Missouri, seemingly along the Mississippi Alluvial Plain that surrounds the Mississippi River. A second large cluster of decreasing prevalence is present in Kansas, Oklahoma and northern Texas. Moving to the western states, the Pacific Coast experienced a mixture of increasing and decreasing prevalence. Very few counties in the Mountain states had significant trends, but those that did were increasing. A large cluster of these trends centers around Colorado, Utah, Arizona and New Mexico.

## Discussion

In the present study, we investigated the changes in canine heartworm infection prevalence from early 2012 through late 2018 at two spatial scales. The interpretations of these two estimates serve different purposes. The regional trend was flexible enough to allow for spatially-varying estimates in different areas of the country so that we did not erroneously assume that dogs in the southern states experienced the same trends as those in northern states. While the regional trends are useful, it is also important to obtain a small-scale view of the changing prevalence, which was done with the local trends.

The ecological fallacy warns that attributes that are inferred from aggregated population data do not necessarily describe the attributes of an individual within that population [15]. In our case, the ecological fallacy warns against drawing conclusions about county-level disease trends from state-level (or any other reasonable spatial aggregation) trends. Moreover, arbitrarily defining regions over which data aggregation should occur is also ill-advised, as different types of aggregation lead to different conclusions. With these concerns in mind, the strength of the proposed statistical construct is that it aptly overcomes both of these issues. Rather than arbitrarily aggregating data over states or other pre-specified regions, our methodology allows the influence of nearby counties on the regional trends to change smoothly with distance. Furthermore, the rate at which the influence decays as distance increases is estimated by the model and is thus driven by the data. Note, the regional trends presented in Fig. 3 represent the group trends, which should be interpreted based on disease patterns across a large geographical area, where one can define the region based on Fig. 2. Smoothing of trends is a common practice in disease mapping [16] and is useful for the global assessment of disease risk and for informing policy. The changes shown here are going to be of most interest to researchers, industry, and government health officials. To reduce (but not entirely eliminate) ecological bias, we separated local temporal trends from the regional trends at the county-level, see Fig. 4a. The local trends clearly demonstrate a different picture. Interpretation of the local trends should only be made in counties with a statistically significant trend, see Fig. 4b. These smaller scale local trends are of interest to veterinarians and pet owners.

Heartworm infections are reported in all states of the USA [1] and the favorable environmental conditions for transmission exist in all states, albeit at different scales [17]. However, the southern United States has long been recognized as an endemic region for canine heartworm infection [18]. Identifying areas that are experiencing the greatest amount of change can help focus future studies aiming to identify the driving factors for these changes. Among the factors to be considered are the densities and ranges of mosquito vector species, wild canid and unprotected domestic dog populations [19, 20], changes in chemoprophylactic drug administration [21], and resistance to the currently available preventative medications [22, 23]. The relative importance of any one factor on prevalence trends is unknown at this time, but the association between any of the aforementioned and the temporal trends discussed here will aid in our understanding of heartworm disease ecology.

### Regional prevalence

The regional temporal trends in *D. immitis* prevalence in the southern states are supported by a similar study conducted by Drake et al. [5]. This study reported temporal trends at the state level (a spatial aggregation between the regional and local trends investigated in the current study). Similar to the regional trends, all the southeastern states studied by Drake et al. were reported to have increased in prevalence with the exception of Mississippi. As discussed below, the reason for this discrepancy is observed in the local trends analysis shown in Fig. 4b, where many of the counties had a negative trend.

The extension of the increasing regional prevalence northwards into Illinois and Indiana is of particular interest and supports the recommendation to increase the use of chemoprophylaxis, preferably year-round, even in states outside of the hyperendemic region.

Florida is an interesting juxtaposition to the surrounding southeastern states. Its similar climate to many of the nearby states would suggest a similar mosquito population and thus exposure to heartworm. One consideration for the regional trends are the many mosquito abatement programs present in Florida [24], particularly southern Florida, in the wake of recent mosquito-borne disease outbreaks [25]. Additionally, Florida may be a success story in terms of preventative care use as *D. immitis* has long been recognized as an important endemic pathogen in Florida, so veterinarians are likely to promote year-round administration and owners may have better compliance.

A lack of increasing regional prevalence outside of the hyperendemic region may be influenced by the same factors discussed above. Fewer mosquito populations and/or lower densities, smaller domestic and wild (e.g. coyote) reservoir populations, shorter transmission periods, and use of preventative medications may reduce the infection pressure enough to prevent a rise in prevalence. As shown in Fig. 1, there are still thousands of dogs testing positive annually outside of the southern states. The presence of stable prevalence trends does not mean there is no risk of infection; that is, dogs are exposed in many parts of the country. Even in regions of low prevalence, the CAPC recommends year-round prevention [1] because the geographical ranges of vectors change [26] and the transmission season is dynamic from year to year [27], making both difficult to predict. Continuous coverage is the best way to protect pets.

### Local prevalence

The local trends (Fig. 4) describe the changes in heartworm infection prevalence at the county level and exhibit a high degree of heterogeneity across the USA. This would be expected as the drivers for local changes may differ from those of the regional trends. Although we cannot infer from this analysis alone, it is possible that the regional trends are driven by long-term changes in the vector population [26], resistance to macrocyclic lactones [9], and climate [28]; while local trends may be driven by shorter-term changes in movement of dogs throughout the USA [29, 30], testing practices of local clinics, availability of different heartworm preventative products [31], expansion of coyote ranges [32], mosquito abatement programs [24], and various landscape factors such as water availability and urban heat islands [33].

Several areas of interest at the local level were found in this study. In particular, increased prevalence occurred in a large cluster of counties throughout Kentucky, Illinois and Indiana; and decreased prevalence occurred in a cluster of counties in Louisiana, Mississippi, Arkansas and Missouri. Both areas deserve further investigation as the identification of the driving factors for both increasing and decreasing prevalence could benefit the control and prevention of heartworm disease nationwide. Increasing local prevalence is also a call to veterinarians to ensure clients are aware of the dangers of heartworm infection and are on appropriate preventatives. Conversely, and importantly, veterinarians in areas with decreasing trends must understand that while prevalence is decreasing as of this study, it may (i) change in the future; and (ii) is only a measure of change in prevalence and does not equate to minimal risk (as baseline prevalence in some areas may remain high).

One posited driving factor that deserves more investigation is the movement of rescue dogs throughout the USA. Large numbers of rescue dogs from the Southeast are transported annually to several states in the Northeast as well as other regions of the USA [30]. Additionally, translocation of stray, shelter and rescue dogs is not unique to the USA [34] and the introduction of non-endemic pathogens into naive populations is a concern worldwide. For this reason, we need to determine if increasing prevalence in historically low prevalence regions are related to the movement of dogs, either through the local testing of those dogs, or the establishment of a reservoir and subsequent autochthonous transmission between local resident dogs. This could be particularly important if relocated dogs are infected with a drug resistant strain of heartworm [29, 35, 36].

Trends unrelated to the domestic dog reservoir include changes in vector habitat suitability [28], temperature and other climate attributes [37], or changes in the density of wildlife reservoirs. Based on the high prevalence observed in field studies, coyotes are highly susceptible to infection [19, 38], and they have only relatively recently become common in parts of the eastern and southern USA [39]. It is possible that changes in the distribution and density of these animals could influence the reservoir pool and subsequently risk of infection in dogs. This could be particularly important as coyotes become more common in urban and suburban areas [40, 41].

Annual and longer-term changes in temperature will alter the length of the heartworm transmission season [42], and so areas practicing seasonal chemoprophylaxis may experience increasing prevalence if chemoprophylaxis is started too late or discontinued too soon to offer full protection. This lapse in coverage may be associated with increased prevalence anywhere throughout the country. Because of this concern and the annual uncertainty in the transmission season, year-round use of chemoprophylaxis is recommended to ensure dogs receive complete coverage [1, 43].

It is critically important for veterinarians and pet owners around the country, particularly in the states with rising heartworm infection prevalence, to assess their current preventative care protocols. Compliance, product efficacy, and the potential for drug-resistant heartworm strains should all be considered. Current recommendations include annual testing for the presence of heartworm antigen and microfilariae, in addition to the year-round preventative medication coverage [1, 43, 44]. Many of the dogs represented by these data are treated and subsequently test negative the following year. Annual testing is especially important in light of the recent evidence of macrocyclic lactone resistance [22, 23]. The areas of significantly positive and negative trends identified in this study should be prioritized in the research efforts of mosquito and *D. immitis* ecology, and in preventative efforts, including pet owner and veterinary professional education. Large-scale studies on the impact of resistance will greatly facilitate our understanding of the rising prevalence of heartworm infection and whether resistance is playing a role in the observed temporal trends.

This analysis is limited by the available data, which was aggregated by month and county and did not contain risk factors at the individual level. Therefore, individual risk of infection cannot be predicted, and even within a single county, risk may have spatial variation. Additionally, these data represent a population of dogs under the care of a veterinarian, and thus, are a conservative estimate of prevalence that may exhibit different temporal dynamics. Local trends are also limited by the available data. At this small scale, it is possible that changes in testing practices within a county (or in the case of large practices, a single clinic) can influence the temporal trend in prevalence. Also, the tested population may not be generalizable to the entire canine population. There are several examples of shelter dogs with significantly higher prevalence compared to owned dogs in the same spatial and temporal units [45, 46]. However, shelter heartworm infection prevalence data are rarely captured at the national level, and prevalence may still be underestimated if shelters are using microfilarial detection *versus* antigen-detection. The use of an antigen-based test for these data has the advantage of better representing newly acquired infections. There are a handful of cases reporting cross-reactivity with heartworm antigen tests from other filarial worm infections, e.g. *Dracunculus* spp., *Angiostrongylus* spp., *Acanthocheilonema* spp. and *Spirocerca* [47–50]. The limited number of reports available for these pathogens in dogs are few, and it is assumed that cross-reactivity is unlikely to influence the reported trends.

The ecology of multi-host, multi-vector pathogens is notoriously difficult to study. Currently, surveillance using testing data such as these is the best way to monitor risk of heartworm infection in domestic dogs. Targeting the vector for heartworm is problematic as there is a large number of vectors in the USA and their distribution and density vary by region as well as habitat [51]. As a result, the relative importance of vectors in local transmission varies. Only known vectors for human pathogens are routinely monitored [26] and so there are few data on other possible vectors of heartworm at the national scale. Even if these data were available, small scale variability would influence risk to individual pets. In addition to studying the spatial importance of vectors, future research should aim to describe the association between heartworm prevalence and temporal trends and the use of chemoprophylaxis and antigen testing. Changes in both testing practices and the use of chemoprophylaxis could have an impact on the trends of infection. Understanding this association could guide testing and prevention recommendations.

## Conclusions

This analysis confirms that heartworm infection is increasing in prevalence in areas throughout the USA. Veterinarians and pet owners (dog, cat and ferret) need to reassess their preventative management of heartworm infection and determine if current protocols are sufficient to protect the animals from infection. More research is needed in determining the major contributing factors, whether it is the impact of climate change or land use on the environment, socioeconomic factors that may impact compliance or access to veterinary care, or pathogen- or vector-related factors that may result in varying mosquito communities or pathogen resistance to medications. Regardless of the cause(s) of the trends we present here, there are actions available to minimize exposure and infection. Year-round preventative medication use is strongly recommended by the CAPC, American Heartworm Society, and the FDA in order to capture any potential changes in the seasonal heartworm transmission window and required annual heartworm infection testing will help detect infections before irreversible pulmonary damage occurs. Routine testing will help document potential drug lack of efficacy events. Additional steps should focus on minimizing mosquito exposure, including the use of dog-approved mosquito repellents and appropriate environmental pesticides, as well as minimizing time outdoors during peak mosquito feeding periods. It is also recommended to use heartworm antigen-detection tests on dogs before travel to historically low prevalence areas of the country, especially in shelter dogs, so that infected dogs are not moved or are treated to minimize risk of introducing heartworm to a new area. Perhaps most importantly, education of pet owners about the increasing risk of heartworm infection will reinforce veterinarian recommendations for prevention and improve compliance.

## Data Availability

The dataset analysed during the current study are available from http://www.capcvet.org.
